# Factors associated with the completion of syphilis treatment among transgender women and travestis, in five Brazilian capitals, 2019-2021: a multicenter cross-sectional study

**DOI:** 10.1590/S2237-96222024v33e2024294.especial.en

**Published:** 2024-12-06

**Authors:** Luis Fernando Gomes Carreira, Maria A. S. Veras, Adele Schwartz Benzaken, Rita Suely Bacuri de Queiroz, Edilene Peres Real Silveira, Elaine Lopes de Oliveira, Katia Cristina Bassichetto, Aline Borges Moreira da Rocha, Bow Suprasert, Erin C. Wilson, Willi McFarland

**Affiliations:** 1 Faculdade de Ciências Médicas, Santa Casa de São Paulo, São Paulo, SP, Brazil; 2 Instituto Leônidas & Maria Deane, Manaus, AM, Brazil; 3 Instituto Adolfo Lutz, São Paulo, SP, Brazil; 4 Núcleo de Pesquisa em Direitos Humanos e Saúde LGBT+, São Paulo, SP, Brazil; 5 Departamento de Saúde Pública de São Francisco, São Francisco, CA, Estados Unidos

**Keywords:** Sífilis, Personas Transgénero, Enfermedades de Transmisión Sexual, Estudios Transversales, Syphilis, Transgender People, Sexually Transmitted Infections, Cross-Sectional Studies

## Abstract

**Objective:**

To assess the previous history of syphilis in transgender women and *travestis* (TWTs) and identify factors associated with treatment incompleteness.

**Methods:**

**:** This was a multicenter cross-sectional study conducted between 2019 and 2021, with participants recruited through respondent-driven sampling, in five Brazilian capitals. Dependent variable: “reported syphilis treatment in the last year”, “no/incomplete” or “complete”. A multivariate-logistic model was used to identify factors associated with completeness.

**Results:**

**:** Of the 1,317 participants, 16.0% reported previous history of syphilis. Of these, 68.9% were Black, 54.6% earned up to 1 minimum wage and 61.1% completed the treatment. Treatment completion was lower in São Paulo (42.7%) and among those who experienced verbal abuse (53.6%; ORa 0.46; 95%CI 0.25;0.85).

**Conclusion:**

In this sample, both the prevalence of self-reported syphilis and the proportion of participants who reported not having started/completed treatment were high. It is essential to identify the barriers faced by TWTs that hinder healthcare access, and identify their needs in order to ensure adequate diagnosis and treatment.

## Study contributions

**Main results:**

Of the 16% of participants who reported a previous diagnosis of syphilis, only one-third completed the treatment. Residents of São Paulo were less likely to complete it compared to those from other cities, and those who had experienced verbal abuse.

**Implications for services:**

There is a need for healthcare professionals to receive training focused on specific needs of TWTs , including respect for their social names, extended opening hours, point-of-care strategy and alternatives for the administration of intramuscular benzathine penicillin injections.

**Perspectives:**

It is crucial to develop new, more effective therapeutic methods for syphilis treatment and deepen research on the impacts of discrimination and stigma on transgender women and *travestis*’ access to healthcare, especially diagnosis and treatment of sexually transmitted infections.

## RESUMEN

**Objetivo:**

Evaluar historia previa de sífilis en mujeres transgénero y travestis (MTT), identificar factores asociados al tratamiento incompleto.

**Métodos:**

Estudio transversal multicéntrico, entre 2019-2021, con participantes reclutadas por Respondent-Driven Sampling, en cinco capitales brasileñas. Variable dependiente: “tratamiento de sífilis reportado el último año”: ‘no/incompleto’ o ‘completo’. Se utilizó un modelo logístico multivariable para identificar factores asociados a la incompletitud.

**Resultados:**

1.317 participantes, 16,0% refirieron sífilis previa. De estas 68,9% eran negras y 54,6% ganaban hasta 1 salario mínimo; 61,1% completaron tratamiento. La finalización del tratamiento fue menor en San Pablo (42,7%) y entre las que sufrieron agresión verbal (53,6%; ORa 0,46; IC95% 0,25;0,85).

**Conclusión:**

Tanto prevalencia de sífilis autorreferida como proporción de participantes que informaron no haber iniciado/finalizado el tratamiento fueron elevadas. Es necesario identificar barreras enfrentadas por MTT que impactan su acceso a atención de salud, además, identificar sus necesidades específicas para garantizar un diagnóstico y tratamiento adecuados.

## INTRODUCTION

Syphilis is one of the most significant Sexually Transmitted Infections ( STIs ) globally and disproportionately affects sexual and gender minorities.^1^ According to data from the Ministry of Health,^2^ in 2023, the detection rate of acquired syphilis in Brazil was 99.2 per 100,000 inhabitants for the general population. However, no information is available on the rates in specific populations affected by the epidemic, such as the lesbian, gay, bisexual, transgender/*travesti* population, since there is no field to record gender identity in the compulsory syphilis notification form. Consequently, estimating the incidence and prevalence of STIs, specifically among transgender women and *travestis* (TWTs ), is challenging and requires data from isolated studies.^1,3,4^ Most studies have found a prevalence of syphilis of approximately one-third in this population in Brazil.^3,4^

The Brazilian transgender population encompasses a range of identities, many of which are still evolving and may change over time, and a portion of transgender women identify with the term “*travestis*” to resignify this socially marginalized gender identity.^5^

The Clinical Protocol and Therapeutic Guidelines (*Protocolo Clínico e Diretrizes Terapêuticas*) for comprehensive care for people with sexually transmitted infections^6^ defines different treatments for syphilis, depending on the disease classification as recent/early (primary, secondary and early latent) or late (late latent, or of unknown duration, and tertiary syphilis). The treatments are available free of charge through the Brazilian National Health System (*Sistema Único de Saúde* – SUS). The treatment of choice consists of intramuscular (IM) injections of benzathine penicillin , in a single dose for recent infections (less than one year), and three doses at weekly interval for late infections. In addition, there is an oral doxycycline regimen, as a second-line treatment reserved for people allergic to penicillin and for those who have prosthetics or industrial liquid silicone at intramuscular injection sites,^6^ which may pose challenges for administration by untrained healthcare professionals.^7^

In Brazil, TWTs face several barriers to accessing adequate treatment. These barriers are related to stigma, discrimination and the limited availability of inclusive healthcare services for the transgender population.^8-10^ This situation directly impact on testing, initiation and the completion of STI treatment .

This study aims to contribute to addressing the knowledge gap regarding the challenges faced by TWTs in completing syphilis treatment. The objective is to evaluate the self-reported previous history of syphilis in TWTs and identify the factors associated with treatment incompleteness.

## METHODS

Data from a cross-sectional study, entitled TransOdara,^11^ conducted between November 2019 and July 2021, with TWTs recruited in capitals of the five Brazilian macro-regions: Campo Grande/Mato Grosso do Sul state, Manaus/Amazonas state, Porto Alegre/Rio Grande do Sul state, Salvador/Bahia state and São Paulo/São Paulo state were used.

The inclusion criteria for participation in the study were as follows: 1) Self-identification as “*travesti*, transgender woman, woman, trans woman or not identifying with the male gender assigned at birth”; 2) Self-reported age: 18 years or older; and 3) Presenting a recruitment coupon provided by a “seed”. The recruitment technique used was respondent *-*driven. sampling (RDS), a suitable approach for reaching hard-to-access populations. Based on participants’ social networks, it assumes that people within a given population are better suited to locate and recruit others from those networks.^12^ In this study, between seven and nine seeds were included per city. There were 22 participant exclusions from the analysis.

In the study, participants responded to a structured questionnaire, which was administered face-to-face by trained interviewers using REDCap . The questionnaire addressed various topics, including: sociodemographic status; experiences with stigma and discrimination; healthcare; prior knowledge about STIs; and testing and treatment for syphilis. Specifically regarding syphilis, participants were asked about and reported on their previous history of infection, initiation and completion of treatment.

For the present study, the outcome variable is “syphilis treatment reported in the last 12 months”, classified as “complete”, measured by a positive response to the question *Have you completed the syphilis treatment?*, and “incomplete”, by a negative response; or “absent”, measured by the negative response to the question *Have you received treatment for syphilis based on your most recent test result?* . Subsequently, the subgroups classified as “incomplete” and “absent” were combined, forming the group “incomplete or absent treatment”, while the subgroup “complete” was termed “complete treatment”. The time frame of the most recent test was measured based on the question *When was your most recent syphilis test?* , considering tests performed in the last 12 months for those who answered “Three months ago”, “Between three and six months” and “Between six months and a year”; and over 12 months for those who answered “Between one and two years” and “More than two years ago”.

The covariates included in the study were: socioeconomic characteristics; healthcare; body modifications; social and interpersonal factors; and sexual behavior. Categorical variables were quantified in absolute frequencies and percentages, while continuous variables were described using means and standard deviations. For analysis purposes only, we combined the subgroups “trans women” and “*travestis*”, using the broader term “trans women”, although it is acknowledged that a portion of the trans population prefers to continue identifying with the term “*travesti”.* The chi-square test was used to compare proportions , with p < 0.05. In the bivariate analysis, only variables with p < 0.20 were considered. In turn, for the multivariate analysis, p < 0.05 was considered, and the variables used for adjustment were: city of recruitment; and prior experience with verbal abuse. The linear regression model was used for multivariate analysis. Chi-square and Fisher’s exact tests were used to test differences in proportions. Student’s *t-* test was used for continuous variables. All analyses were performed using the Stata, version 14.0.

Responses such as “don’t know”, “refused to answer” or “not applicable” were coded as missing for positive results for syphilis, according to: sexual orientation (n = 1); race/skin color (n = 2); occupation (n = 2); monthly income (n = 17); consultation with a healthcare professional (n = 1); mistreated by a healthcare professional (n = 1); verbal abuse in the last 12 months (n = 3); physical aggression in the last 12 months (n = 1); first consensual sexual relationship (n = 5).

Responses such as “not applicable” were coded as “no”. Responses such as “don’t know” or “refused to answer” were coded as missing for positive results for syphilis, according to: incarceration (n = 1); consultation with a healthcare professional in the last 12 months (n = 1); exchanged sex for goods (n = 2); dating apps (n = 3).

Responses such as “don’t know” or “not applicable” were coded as “unknown”. Responses such as “refused to answer” were coded as missing, for positive results for syphilis, based on HIV serology (n = 3).

Responses such as “don’t know”, “refused to answer” or “not applicable” were coded as missing for negative results for syphilis, according to: city (n = 1); sexual orientation (n = 3); race/skin color (n = 3); occupation (n = 1); monthly income (n = 23); use of industrial silicone (n = 4); first consensual sexual relationship (n = 11).

Responses such as “not applicable” were coded as “no”. Responses such as “don’t know” or “refused to answer” were coded as missing for negative results for syphilis, according to: incarceration (n = 1); consultation with a healthcare professional in the last 12 months (n = 3); exchanged sex for goods (n = 119); use of dating apps (n = 10).

The project was approved by the Research Ethics Committee (REC) of Santa Casa de Misericórdia de São Paulo (CAAE: 05585518.7.0000.5479). In addition, it received approval from other RECs involved. Participants provided written informed consent and were assured of referrals to clinical and social service providers when necessary.

## RESULTS

**Figure 1:**
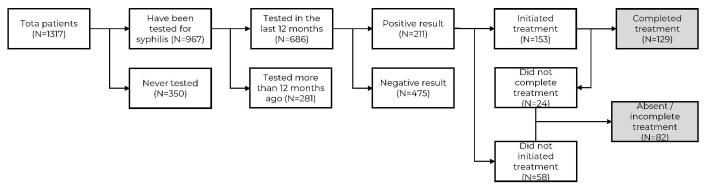
Care cascade for self-reported syphilis testing and treatment among transgender women and *travestis* in five Brazilian cities, 2019-2021 (n = 1,317)

Of the 1,317 TWTs recruited, 967 (73.4%) reported having been tested for syphilis, with 686 (70.9%) in the last 12 months. Of these, 475 (69.3%) had a negative result, while 211 (30.7%) reported a positive test, with 82/211 (38.9%) of them reporting not initiating or completing treatment, and 129/211 (61.1%) reporting completing it (Figure 1).

The socioeconomic and demographic characteristics of the 686 TWTs who reported syphilis testing in the last 12 months are shown in Table 1. Regarding the 211 TWTs who reported testing positive: the majority were from São Paulo (45.5%), followed by Manaus (21.3%) and Salvador (13.3%). Young people aged 18 to 24 years comprised 16.1% of the sample, while people aged 30 to 39 years accounted for 36.3%. The majority identified as transgender women (60.2%) and heterosexual (84.3%). The average age at first sexual intercourse was 12.5 years (SD = 3.5) and 84.0% of them declared that it was a consensual relationship.

**Table 1:** Comparison of sociodemographic characteristics of transgender women and travestis, according to reported syphilis testing results in the last 12 months, in five Brazilian capitals, TransOdara study, 2019-2021 (n = 686)

Variables	Positive syphilis result in the last 12 months n = 211 (%)	Negative syphilis result in the last 12 months n = 475 (%)	p-value
Sociodemographic characteristics			
**City**			0.391
São Paulo	96 (45.5)	203 (42.8)	
Porto Alegre	25 (11.9)	63 (13.3)	
Savior	28 (13.3)	63 (13.3)	
Manaus	45 (21.3)	85 (17.9)	
Campo Grande	17 (8.1)	60 (12.7)	
Missing	0	1	
**Age group (years)**			0.051
18-24	34 (16.1)	123 (25.9)	
25-29	50 (23.7)	111 (23.4)	
30-39	76 (36.0)	135 (28.4)	
40-49	38 (18.0)	74 (15.6)	
50+	13 (6.2)	32 (6.7)	
**Gender identity**			0.808
Woman	16 (7.6)	36 (7.6)	
Transgender woman	127 (60.2)	295 (62.1)	
*Travesti*	62 (29.4)	121 (25.5)	
Transsexual	2 (1.0)	11 (2.3)	
Non-binary	3 (1.4)	9 (1.9)	
Others	1 (0.5)	2 (0.6)	
**Sexual orientation**			0.031
Heterosexual	177 (84.3)	360 (76.3)	
Homosexual	8 (3.8)	33 (7.0)	
Bisexual	17 (8.1)	30 (6.4)	
Pansexual	8 (3.8)	44 (9.3)	
Asexual	0	1 (0.2)	
Others	0	4 (0.9)	
Missing	1	3	
**Race/skin color**			0.757
White	58 (27.8)	128 (27.1)	
Black	48 (23.0)	121 (25.6)	
Asian	3 (1.4)	13 (2.8)	
Mixed-race	96 (45.9)	201 (42.6)	
Indigenous	4 (1.9)	9 (1.9)	
Missing	2	3	
**Education level**			0.000
Elementary school or less	70 (33.2)	89 (18.7)	
Incomplete high school	37 (17.5)	78 (16.4)	
Complete high school	73 (34.6)	182 (38.3)	
Higher education or more	31 (14.7)	126 (26.5)	
**Occupation**			0.011
Formal employment	57 (27.3)	136 (28.7)	
Informal employment	43 (20.6)	125 (26.4)	
Unemployed	37 (17.7)	100 (21.1)	
Student	9 (4.3)	27 (5.7)	
Sex worker	63 (30.1)	86 (18.1)	
Missing	2	1	
**Housing situation**			0.861
Private or rented	139 (65.9)	317 (66.7)	
Temporary housing (family, friends or work)	46 (21.8)	102 (21.5)	
Shelter or homeless	16 (7.6)	29 (6.1)	
Other	10 (4.7)	27 (5.7)	
**Monthly income**			0.328
Up to 1 minimum wage	106 (54.6)	228 (50.4)	
Above the minimum wage	88 (45.4)	224 (49.6)	
Missing	17	23	
**Incarceration**			0.000
No	133 (63.3)	373 (78.7)	
Yes	77 (36.7)	101 (21.3)	
Missing	1	1	
**Healthcare**			0.000
HIV serology			
Negative or unknown	138 (66.4)	376 (79.2)	
Positive	70 (33.7)	99 (20.8)	
Missing	3	0	
**Consultation with a healthcare professional in the last 12 months**			0.831
No (no need)	32 (15.2)	64 (13.6)	
No (there was a need)	8 (3.8)	17 (3.6)	
Yes	170 (81.0)	391 (82.8)	
Missing	1	3	
**HIV test in the last 12 months**			0.695
No	34 (16.1)	71 (14.9)	
Yes	177 (83.9)	404 (85.1)	
**Body modifications**			
**Use of hormones for gender transition**			0.24
No	15 (7.1)	47 (9.9)	
Yes	196 (92.9)	428 (90.1)	
**Use of industrial silicone**			0.001
No	127 (60.2)	345 (73.2)	
Yes	84 (39.8)	126 (26.8)	
Missing	0	4	
**Gender-affirming surgery**			0.663
No	208 (98.6)	466 (98.1)	
Yes	3 (1.4)	9 (1.9)	
**Social and interpersonal factors**			
**Discrimination in the last 12 months**			0.837
No			
Yes			
Missing			
**Mistreated by healthcare professionals**			0.791
No	141 (67.3)	314 (66.1)	
Yes	69 (32.7)	161 (33.9)	
Missing	1	0	
**Verbal abuse, humiliation or insult in the last 12** **months**			0.968
No	98 (47.1)	223 (46.9)	
Yes	110 (52.9)	252 (53.1)	
Missing	3	0	
Physical aggression in the last 12 months			0.064
No	165 (78.6)	400 (84.4)	
Yes	45 (21.4)	74 (15.6)	
Missing	1	1	
**Sexual behavior**			
**Sex work as a source of income in the last month**			0.004
No	140 (66.3)	365 (76.8	
Yes	71 (33.7)	110 (23.2)	
**Engaged in sex in exchange for goods**			0.036
No	114 (25.8)	128 (36.0)	
Yes	95 (74.2)	228 (64.0)	
Missing	2	119	
**Use of dating apps**			0.337
No	143 (68.1)	299 (64.3)	
Yes	65 (31.9)	166 (35.7)	
Missing	3	10	
**First sexual intercourse**			0.471
No	33 (16.0)	85 (18.3)	
Yes	173 (84.0)	379 (81.7)	
Missing	5	11	

Analyzing sociodemographic characteristics: 65.9% of people reported living in their own or rented house or apartment and 54.6% had an income of up to 1 minimum wage^13^ (in 2021, BRL 1,100.00). Regarding race/skin color, 45.9% as mixed-race and 23.0% as Black. The highest level of education was complete high school (for 34.6% of the sample), and sex work was reported as the main occupation for 30.1% of the sample. About one-third (36.7%) had been incarcerated. As for health care, 81.0% consulted a healthcare professional in the last 12 months, 83.9% were tested for HIV, and 33.7% tested positive for HIV. With regard to body modification, 92.6% used hormones for gender transition, 39.8% used industrial silicone and 1.4% had undergone gender-affirming surgery. Approximately 90.0% reported experiencing discrimination in the last 12 months, 52.1% experienced verbal abuse and 21.3% were physically assaulted for being TWT . It is worth highlighting that 32.7% reported having been mistreated by healthcare professionals at some point.

There was a statistically significant difference between the groups that reported positive and negative syphilis test results in the following variables: sexual orientation, schooling, occupation, incarceration, HIV serology, use of industrial silicone, sex work as a source of income in the last month and exchanging sex for goods. Other characteristics are described in Table 1.

Table 2 shows the prevalence of sociodemographic and behavioral characteristics resulting from the bivariate analysis (OR) and the adjusted multiple model (ORa ) for each subgroups reporting a positive testing (absent/incomplete treatment and complete treatment).

**Table 2:** Bivariate and multivariate analysis, (OR) and (ORa), with 95% confidence intervals (95%CI), independent associations with self-reported syphilis treatment completion among transgender women and travestis in five Brazilian capitals, 2019-2021 (n = 211)

Variables	Incomplete or absent treatment	Complete treatment	p-value	OR (95%CI)	ORa (95%CI)
	n = 82 (38.9%)	n = 129 (61.1%)			
**Sociodemographic characteristics**					
**City**							
São Paulo	55 (57.3)	41 (42.7)	< 0.001	ref.	ref.
Porto Alegre	5 (20.0)	20 (80.0)		5.37 (1.86;15.49)	5.89 (2.00;17.34)
Salvador	9 (32.1)	19 (67.9)		2.83 (1.16;6.90)	3.09 (1.24;7.66)
Manaus	8 (17.8)	37 (82.2)		6.20 (2.61;14.73)	6.25 (2.60;15.03)
Campo Grande	5 (29.4)	12 (70.6)		3.22 (1.05;9.86)	3.73 (1.19;11.70)
**Age group (years)**							
18-24	13 (38.2)	21 (61.8)	0.693		
25-29	20 (40.0)	30 (60.0)			
30-39	32 (42.1)	44 (57.9)			
40-49	11 (29.0)	27 (71.1)			
50+	6 (46.2)	7 (53.9)			
**Gender identity**					
Woman	9 (56.3)	7 (43.8)	0.276		
Transgender woman	52 (40.9)	75 (59.1)			
*Travesti*	21 (33.9)	41 (66.1)			
Transsexual	0	2 (100.0)			
Non-binary	0	3 (100.0)			
Others	0	1 (100.0)			
**Sexual orientation**					
Heterosexual	73 (41.2)	104 (58.8)	0.589		
Homosexual	2 (25.0)	6 (75.0)			
Bisexual	5 (29.4)	12 (70.6)			
Pansexual	2 (25.0)	6 (75.0)			
Missing	0	1			
**Race/skin color**					
White	22 (37.9)	36 (62.1)	0.986		
Black	18 (37.5)	30 (62.5)			
Asian	1 (33.3)	2 (66.7)			
Mixed-ra	38 (39.6)	58 (60.4)			
Indigenous	2 (50.0)	2 (50.0)			
Missing	1	1			
**Education level**					
Elementary school or less	25 (35.7)	45 (64.3)	0.498		
Incomplete high school	18 (48.7)	19 (51.4)			
Complete high school	29 (39.7)	44 (60.3)			
Higher education or more	10 (32.3)	21 (67.8)			
**Occupationz**							
Formal employment	19 (33.3)	38 (66.7)	0.105	ref.
Informal employment	13 (33.3)	26 (66.7)		1.00 (0.42;2.37)
Unemployed	13 (31.7)	28 (68.3)		1.08 (0.46;2.54)
Student	6 (66.7)	3 (33.3)		0.25 (0.06;1.11)
Sex worker	31 (49.2)	32 (50.8)		0.52 (0.25;1.08)
Missing	0	2			
**Housing situation**							
Private or rented	60 (43.2)	79 (56.8)	0.307		
Temporary housing (family, friends or work)	14 (29.8)	33 (70.2)			
Shelter or homeless	6 (37.5)	10 (62.5)			
Other	2 (22.2)	7 (77.8)			
**Monthly income**							
Up to 1 minimum wage	39 (36.8)	67 (63.2)	0.456		
Above 1 minimum wage	37 (42.1)	51 (58.0)			
Missing	6	11			
**Incarceration**							
No	52 (39.1)	81 (60.9)	0.837		
Yes	29 (37.7)	48 (62.3)			
Missing	1	0			
**Health care**							
**HIV** **serology**							
Negative or unknown	61 (44.2)	77 (55.8)	0.029	ref.
Positive	20 (28.6)	50 (71.4)		1.98 (1.07;3.67)
Missing	1	2		
**Consultation with a healthcare professional in the last 12** **months**							
No (no need)	15 (46.9)	17 (53.1)	0.479		
No (there was a need)	2 (25.0)	6 (75.0)			
Yes	64 (37.7)	106 (62.4)			
Missing	1	0			
**HIV test in the last 12 months**							
No	7 (20.6)	27 (79.4)	0.017	ref.
Yes	75 (42.4)	102 (57.6)		0.35 (0.15;0.85)
**Body modifications**							
**Use of hormones for gender transition**							
No	6 (40.0)	9 (60.0)	0.925		
Yes	76 (38.8)	120 (61.2)			
**Use of industrial silicone**							
No	43 (33.9)	84 (66.1)	0.067	ref.
Yes	39 (46.4)	45 (53.6)		0.59 (0.34;1.04)
**Gender-affirming surgery**							
No	82 (39.4)	126 (60.6)	0.164		
Yes	0	3 (100.0)			
**Social and interpersonal factors**							
**Have you experienced discrimination in the last 12 months?**							
No	12 (46.2)	14 (53.9)	0.415		
Yes	70 (37.8)	115 (62.2)			
**Have you ever been mistreated by healthcare professionals?**							
No	55 (38.7)	87 (61.3)	0.956		
Yes	27 (39.1)	42 (60.9)			
**Verbally assaulted, humiliated or insulted in the last 12 months**							
No	31 (30.7)	70 (69.3)	0.020	ref.	ref.
Yes	51 (46.4)	59 (53.6)		0.51 (0.29;0.90)	0.46 (0.25;0.85)
**Have you been physically assaulted in the last 12 months?**							
No	61 (36.8)	105 (63.3)	0.226		
Yes	21 (46.7)	24 (53.3)			
Sexual behavior							
**Sex work as a source of income in last month**							
No	50 (61.0)	90 (69.8)	0.188		
Yes	32 (45.1)	39 (54.9)			
**Engaged in sex in exchange for money, goods, drugs, or a place to live**							
No	51 (44.7)	63 (55.3)	0.074	ref.
Yes	31 (32.6)	64 (67.4)		1.67 (0.95;2.94)
Missing	0	2			
**Use of dating apps**							
No	57 (39.9)	86 (60.1)	0.539		
Yes	23 (35.4)	42 (64.6)			
Missing	2	1			
**First consensual sexual intercourse**							
No	13 (39.4)	20 (60.6)	0.992		
Yes	68 (39.3)	105 (60.7)			
Missing	1	2			

The following factors were associated with the outcome in the bivariate analysis: having been tested for HIV and having tested positive for HIV in the past 12 months, city of recruitment, and experience of verbal abuse for being a transgender woman. Participants in São Paulo reported substantially lower levels of treatment completion compared to other cities (São Paulo, 42.7%; Salvador, 67.9%; Campo Grande, 70.6%; Porto Alegre, 80.0%; and Manaus, 82.7%, p < 0.001).

TWTs who reported a positive HIV test were more likely to complete syphilis treatment compared to those who reported a negative HIV test or unknown status (71.4% vs. 55.8%, p = 0.029). TWTs tested for HIV in the past 12 months were less likely to complete syphilis treatment compared to those who were not tested (57.6% *vs.* 79.4%, p = 0.017).

Based on the adjusted multiple model, only the study city and previous experience of verbal abuse remained associated with incompleteness/absence of syphilis treatment. Compared to TWTs in São Paulo, participants from other cities showed higher completion rates, ranging from ORa 3.09 (95%CI 1.24;7.66) in Salvador to ORa 6.25 (95%CI 2.60;15.03) in Manaus. TWTs who were verbally abused, humiliated or insulted for being TWT in the last 12 months were less likely to complete treatment (ORa 0.46; 95%CI 0.25;0.85).

## DISCUSSION

The study revealed a concerning history of syphilis among TWTs, exacerbated by low treatment adherence. More than one-third of participants with a prior diagnosis of syphilis reported not having initiated or completed treatment. This high rate of treatment absence/incompleteness is similar to that found in Lima, Peru, where 31.3% of TWTs and men who have sex with men (MSM), after testing positive for syphilis, faced similar challenges.^14^

Regarding associated factors, surprisingly, participants from São Paulo had the lowest rate of treatment completion, despite it being the largest city in the country and located relatively wealthier Southeast region, which is relatively wealthier. On the other hand, in Manaus, despite more limited financial resources, participants had the highest level of treatment adherence. We believe that the existence of an outpatient clinic specialized in sexual and gender diversity in Manaus, which facilitates healthcare access for this population, may help explain a higher rate in the city compared to São Paulo. Although São Paulo has a broader health care network, it offers heterogeneous experiences in treating transgender people, which may influence the different rates of syphilis treatment completion.

It is worth highlighting that the experience of verbal abuse, especially for TWTs , is associated with a lower likelihood of completing syphilis treatment. This occurs because TWTs often face high levels of interpersonal violence of various types.^15^ Verbal abuse may be an indicator of the widespread discrimination faced by TWTs in Brazil;^16-18^ furthermore, it is well-documented that TWTs may avoid seeking healthcare out of fear of discrimination in the SUS.^8,9^ Hesitancy in using healthcre services to obtain diagnosis and treatment for syphilis and other STIs among TWTs is a strategy employed to avoid the stigma that often occurs in these settings.^9^

Another plausible hypothesis for low treatment adherence during the analyzed period may be related to the Covid-19 pandemic, which impacted healthcare access for vulnerable groups, including TWTs.^19^

The Covid-19 pandemic, although delaying interviews due to the suspension of fieldworks, owing to curfews and measures to reduce the circulation of people during the restriction period, in all cities studied, did not hinder our ability to reach and even exceed the initially calculated sample size.

Barriers to syphilis treatment among TWTs are present at all stages of the care cascade. To confirm the diagnosis, two tests are required, one treponemal and one non- treponemal , which are generally not performed simultaneously.^6^

Although it is currently recommended that treatment for people from more vulnerable groups be initiated immediately after a positive rapid test result, this guidance may not be widely known or followed by all healthcare professionals.^6^ In addition, the complexity of the treatment may also be a barrier, given that, diagnosis of late syphilis, requires penicillin injections at seven-day intervals, or oral doxycycline treatment, which must be maintained for one month.^6^

Another factor that influences the care cascade is the difficulty in tracing sexual partners for treatment, and the challenge of negotiating the use of condoms as a preventive method with commercial partners, as documented in MSM groups.^20^

Additionally, the use of industrial liquid silicone, a practice still common in this group,^21^ makes intramuscular administration of penicillin difficult. This is because it becomes necessary to inject into alternative sites, such as the ventrogluteal , which healthcare professionals are less familiar with.^7^ Alternative treatment with doxycycline , often recommended for TWTs with industrial silicone, may lead to treatment abandonment, due to low tolerability, side effects (gastrointestinal), prolonged treatment duration, dosage (twice a day) and potential drug interactions.^6^

Other structural factors may contribute to the difficulty of appropriately treating TWTs, and include lack of accessibility, unwelcoming services,^8-10^ being victims of harassment on transportation,^22^ lack of resources for transportation^23^ or even the opening hours of healthcare services,^23^ which are incompatible with the working hours of some of them.

The different rates of treatment incompleteness/absence across cities call for more detailed studies investigating the reasons for these findings, in order to guide actions aimed at mitigating treatment incompleteness and reducing the high prevalence of syphilis in this population.

Healthcare professionals, managers, multidisciplinary teams and civil society in Brazil need to be more aware of the care of transgender people and *travestis*, and involved in reducing the barriers they face in the healthcare system. This is especially important for sexual health, risk behaviors and screening for syphilis and other STIs . Although incomplete/absent treatment for syphilis was not associated with aggression by health professionals in this sample, nearly one-third of participants reported such experiences in the last year, corroborating the literature.^24^

This study has several limitations. First, the data were obtained through self-report, which is subject to recall bias. Second, the data are not representative of the total number of TWTs in Brazil. Although the RDS is widely used to study populations considered hard-to-reach, it is important to interpret these results with caution, as they may only represent the social networks captured by the study in each of the cities studied, excluding networks from other states or smaller cities in the same state. Despite the limitations mentioned, the study recruited a significant number of TWTs across various regions of Brazil, and its findings contribute to the growing literature on syphilis in transgender women and *travestis*, being one of the few studies that examined treatment for syphilis in this population group.

Routine screening and treatment for syphilis need to be integrated into health programs nationwide. New technologies, such as molecular diagnostic methods, self-testing,^25^ peer-based strategies,^26^ and flexible healthcare service hours,^23^ could help mitigate these barriers, and should include an evaluation of the effectiveness of these different options. In addition, healthcare professionals should be aware of the legislation and protocols that have already been established to meet the needs of this population, including the use of the social name for TWTs. in healthcare settings, administering injectable medication in alternative sites and establishing mechanisms to prevent and address discrimination faced by TWTs in healthcare settings.^9^
