# Supplementary material for: Factors associated with the completion of syphilis treatment among transgender women and travestis, in five Brazilian capitals, 2019-2021: a multicenter cross-sectional study
Source: Epidemiol Serv Saude. 2024 Dec 6;33(spe1):e2024294. doi: 10.1590/S2237-96222024v33e2024294.especial.en (PMC11654392; doi:10.1590/S2237-96222024v33e2024294.especial.en)
Supplement: Translation

# Translation: Fatores associados à completude do tratamento de sífilis em mulheres trans e travestis, em cinco capitais brasileiras, 2019-2021: estudo multicêntrico transversal

**DOI:** 10.1590/S2237-96222024v33e2024294.especial.pt

**Authors:** Luis Fernando Gomes Carreira, Maria A. S. Veras, Adele Schwartz Benzaken, Rita Suely Bacuri de Queiroz, Edilene Peres Real Silveira, Elaine Lopes de Oliveira, Katia Cristina Bassichetto, Aline Borges Moreira da Rocha, Bow Suprasert, Erin C. Wilson, Willi McFarland

**Affiliations:** 1 Faculdade de Ciências Médicas, Santa Casa de São Paulo, São Paulo, SP, Brasil; 2 Instituto Leônidas & Maria Deane, Manaus, AM, Brasil; 3 Instituto Adolfo Lutz, São Paulo, SP, Brasil; 4 Núcleo de Pesquisa em Direitos Humanos e Saúde LGBT+, São Paulo, SP, Brasil; 5 Departamento de Saúde Pública de São Francisco, São Francisco, CA, Estados Unidos

**Keywords:** Sífilis, Pessoas Transgênero, Infecções Sexualmente Transmissíveis, Estudos Transversais

## Abstract

**Objetivo:**

Avaliar a história pregressa de sífilis em mulheres trans e travestis (MTTr) e identificar os fatores associados à incompletude do tratamento.

**Métodos:**

Estudo transversal multicêntrico, entre 2019 e 2021, com participantes recrutadas por *respondent-driven sampling*, em cinco capitais brasileiras. Variável dependente: “tratamento referido de sífilis no último ano”, “não/incompleto” ou “completo”. Modelo multivariável-logístico foi utilizado para identificar fatores associados à completude.

**Resultados:**

Das 1.317 participantes, 16,0% relataram sífilis pregressa. Dessas, 68,9% eram negras, 54,6% ganhavam até 1 salário mínimo e 61,1% completaram o tratamento. A completude de tratamento foi mais baixa em São Paulo (42,7%) e para quem sofreu agressão verbal (53,6%; ORa 0,46; IC_95_% 0,25;0,85).

**Conclusão:**

Na amostra, foram elevadas: a prevalência de sífilis autorreferida e a proporção de participantes que refere não ter iniciado/completado o tratamento. É necessário identificar as barreiras enfrentadas por MTTr que impactam o acesso à saúde, e identificar suas necessidades, para assegurar diagnóstico e tratamento adequados.

## Contribuições do estudo

**Principais resultados:**

Das 16% das participantes que relataram diagnóstico de sífilis pregressa, apenas um terço completaram o tratamento. Tiveram menor probabilidade de completá-lo as residentes de São Paulo, em comparação às de outras cidades, e aquelas que sofreram abuso verbal.

**Implicações para os serviços:**

Necessidade de formação de profissionais de saúde direcionada a necessidades específicas de MTTr, incluindo respeito ao nome social, ampliação de horário de atendimento, estratégia de *point-of-care* e alternativas para a aplicação de injeções intramusculares de penicilina benzatina.

**Perspectivas:**

Desenvolver novos métodos terapêuticos mais efetivos para o tratamento da sífilis e aprofundar as pesquisas sobre os impactos da discriminação e o estigma na busca de mulheres trans e travestis ao acesso à saúde, em especial, diagnóstico e tratamento de infecções sexualmente transmissíveis.

## INTRODUÇÃO

A sífilis é uma das Infecções Sexualmente Transmissíveis (ISTs) mais relevantes globalmente e afeta desproporcionalmente minorias sexuais e de gênero.^1^ Segundo dados do Ministério da Saúde,^2^ em 2023, a taxa de detecção de sífilis adquirida no Brasil foi de 99,2 por 100 mil habitantes para a população em geral. Entretanto, não há informações sobre as taxas em populações específicas para a epidemia, como a população de lésbicas, *gays*, bissexuais, transexuais/travestis (LGBT), já que não há campo para registrar a identidade de gênero na ficha de notificação compulsória de sífilis. Em função disso, há dificuldade de estimar incidência e prevalência de ISTs, especificamente entre mulheres trans e travestis (MTTr), sendo necessário obter essas informações a partir de estudos isolados.^1,3,4^ A maioria dos estudos encontrou uma prevalência de sífilis em torno de um terço nessa população no Brasil.^3,4^

A população de pessoas transsexuais brasileira abrange uma variedade de identidades ainda em construção e que podem ser modificadas ao longo do tempo, e uma parte da população trans feminina opta por se identificar com o termo “travesti” para ressignificar essa identidade de gênero socialmente marginalizada.^5^

O Protocolo Clínico e Diretrizes Terapêuticas para atenção integral a pessoas com infecções sexualmente transmissíveis^6^ define diferentes tratamentos para sífilis, a depender da classificação da doença em recente/precoce (primária, secundária e latente recente) ou tardia (latente tardia, ou de duração ignorada, e sífilis terciária). Os tratamentos estão disponíveis gratuitamente no Sistema Único de Saúde (SUS). O tratamento de eleição consiste em injeções intramusculares (IM) de penicilina benzatina, em dose única para as infecções recentes (menos de um ano), e três doses para a tardia, com intervalo semanal. Além disso, há também tratamento por via oral com doxiciclina, de segunda linha e reservado para pessoas alérgicas à penicilina e para aquelas que tenham próteses ou silicone líquido industrial nos locais de aplicação intramuscular,^6^ dificultando a aplicação por profissionais não treinados.^7^

No Brasil, as MTTr enfrentam diversas barreiras para acessar tratamento adequado. Essas barreiras estão relacionadas ao estigma, à discriminação e à pouca oferta de serviços de saúde inclusivos para a população trans.^8-10^ Essa situação impacta diretamente a testagem, o início e a conclusão do tratamento de ISTs.

Este estudo tem como propósito contribuir para preencher a lacuna de conhecimento acerca das dificuldades enfrentadas pelas MTTr na conclusão do tratamento da sífilis. O objetivo é avaliar a história pregressa autorrelatada de sífilis em MTTr e identificar os fatores associados à incompletude do tratamento.

## MÉTODOS

Foram utilizados dados advindos de uma pesquisa transversal, denominada TransOdara,^11^ realizada entre novembro de 2019 e julho de 2021, com MTTr recrutadas em capitais das cinco macrorregiões brasileiras: Campo Grande/MS, Manaus/AM, Porto Alegre/RS, Salvador/BA e São Paulo/SP.

Os critérios de inclusão para participação no estudo foram os seguintes: 1) Autoidentificação como “travesti, mulher transexual, mulher, mulher trans ou não se identificar com o gênero masculino designado ao nascimento”; 2) Idade autorreferida: ter 18 anos ou mais; e 3) Apresentar cupom de recrutamento fornecido por uma “semente”. A técnica de recrutamento foi a *respondent-driven sampling* (RDS), abordagem adequada para alcançar populações de difícil acesso. Baseada nas redes sociais das participantes, pressupõe que pessoas de uma determinada população são melhores para localizar e recrutar outras pessoas dessas redes.^12^ Neste estudo, entre sete e nove sementes foram incluídas por cidade. Houve 22 exclusões de participantes da análise.

No estudo, as participantes responderam a um questionário estruturado, que foi administrado presencialmente por entrevistadores(as) treinados(as), utilizando-se o REDCap. O questionário abordou diversos tópicos, incluindo: situação sociodemográfica; experiências com estigma e discriminação; cuidados com a saúde; conhecimento prévio sobre IST; e testagem e tratamento de sífilis. Especificamente em relação à sífilis, as participantes foram questionadas e relataram sobre a história prévia de infecção, início e completude do tratamento.

Para o presente estudo, a variável de desfecho é o “tratamento de sífilis referido nos últimos 12 meses”, classificado como “completo”, medido pela resposta positiva à pergunta *Você terminou o tratamento para sífilis?*, e “incompleto”, pela negativa; ou “ausente”, medido pela resposta negativa à pergunta *Você recebeu tratamento para sífilis em função do resultado do seu teste mais recente?*. Posteriormente, os subgrupos classificados como “incompleto” e “ausente” foram somados, passando a se denominar como “tratamento incompleto ou ausente”, enquanto o subgrupo “completo” foi denominado “tratamento completo”. A definição sobre o tempo do teste mais recente foi medida a partir da pergunta *Quando foi seu teste mais recente para sífilis?*, considerando-se como tendo sido realizado nos últimos 12 meses para aquelas que responderam “Há três meses”, “Entre três e seis meses” e “Entre seis meses e um ano”; e acima dos 12 meses para aquelas que responderam “Entre um e dois anos” e “Há mais de dois anos”.

As covariáveis incluídas no estudo foram: características socioeconômicas; cuidados de saúde; modificações corporais; fatores sociais e interpessoais; comportamento sexual. As variáveis categóricas foram quantificadas em frequências absolutas e percentuais, enquanto as variáveis contínuas foram descritas por médias e desvios- padrão. Exclusivamente para fins da análise, agregamos os subgrupos “mulheres trans” e “travestis”, utilizando o termo mais amplo “mulheres trans”, embora se reconheça que uma parte da população trans opte por continuar se identificando com o termo *travesti*. Para as comparações de proporções, foi utilizado o teste qui-quadrado, com p < 0,05. Na análise bivariada, foram consideradas apenas variáveis com p < 0,20. Por seu turno, na multivariada foram considerados p < 0,05, e as variáveis usadas para ajuste foram: cidade de recrutamento; e experiência prévia com abuso verbal. O modelo de regressão linear foi utilizado para análise multivariada. Os testes qui quadrado e exato de Fisher foram usados para testar diferenças nas proporções. O teste *t* de Student foi usado para variáveis contínuas. Todas as análises foram realizadas utilizando-se o Stata, versão 14.0.

Respostas “não sei”, “recusou-se a responder” ou “não se aplica” foram codificadas como *missing* para resultados positivos para sífilis, segundo: orientação sexual (n = 1); raça/cor da pele (n = 2); ocupação (n = 2); renda mensal (n = 17); consulta com profissional de saúde (n = 1); maltratadas por profissional de saúde (n = 1); agressão verbal nos últimos 12 meses (n = 3); agressão física nos últimos 12 meses (n = 1); primeira relação sexual consentida (n = 5).

Respostas “não se aplica” foram codificadas como “não”. Respostas “não sei” ou “recusou-se a responder” foram codificadas como *missing* para resultados positivos para sífilis, segundo: encarceramento (n = 1); consulta com profissional de saúde nos últimos 12 meses (n = 1); fez sexo em troca de bens (n = 2); aplicativos de relacionamento (n = 3).

Respostas “não sei” ou “não se aplica” foram codificadas como “desconhecidos”. Respostas “recusou-se a responder” foram codificadas como *missing* para resultados positivos para sífilis, segundo sorologia de HIV (n = 3).

Respostas “não sei”, “recusou-se a responder” ou “não se aplica” foram codificadas como *missing* para resultados negativos para sífilis, segundo: cidade (n = 1); orientação sexual (n = 3); raça/cor da pele (n = 3); ocupação (n = 1); renda mensal (n = 23); uso de silicone industrial (n = 4); primeira relação sexual consentida (n = 11).

Respostas “não se aplica” foram codificadas como “não”. Respostas “não sei” ou “recusou-se a responder” foram codificadas como *missing* para resultados negativos para sífilis, segundo: encarceramento (n = 1); consulta com profissional de saúde nos últimos 12 meses (n = 3); fez sexo em troca de bens (n = 119); uso de aplicativos de relacionamento (n = 10).

O projeto foi aprovado pelo Comitê de Ética em Pesquisa (CEP) da Santa Casa de Misericórdia de São Paulo (CAAE: 05585518.7.0000.5479). Além disso, obteve aprovação de outros CEPs envolvidos. As participantes forneceram consentimento por escrito e foram assegurados encaminhamentos para serviços clínicos e de serviço social, quando necessário.

## RESULTADOS

Das 1.317 MTTr recrutadas, 967 (73,4%) relataram já terem se testado para sífilis, sendo 686 (70,9%) nos últimos 12 meses. Dessas, 475 (69,3%) tiveram resultado negativo, enquanto 211 (30,7%) relataram um teste positivo, sendo que 82/211 (38,9%) delas relataram não iniciar ou não completar o tratamento, e 129/211 (61,1%) relataram concluí-lo (Figura 1).

**Figura 1:** Cascata de cuidados de testagem e tratamento autorrelatado de sífilis entre mulheres trans e travestis em cinco cidades brasileiras, 2019-2021 (n = 1.317)

As características socioeconômicas e demográficas das 686 MTTr com relato de testagem para sífilis nos últimos 12 meses estão apresentadas na Tabela 1. Sobre as 211 MTTr que relataram testar positivo; a maioria era de São Paulo (45,5%), vindo em seguida Manaus (21,3%) e Salvador (13,3%). Jovens de 18 a 24 anos compreendiam 16,1% da amostra, enquanto pessoas de 30 a 39 anos representaram 36,3%. A maioria se autodeclarava mulher trans (60,2%) e heterossexual (84,3%). A idade média na primeira relação sexual foi de 12,5 anos (DP = 3,5) e 84,0% delas declararam que se tratou de uma relação consensual. 

**Tabela 1:** Comparação das características sociodemográficas de mulheres trans e travestis, segundo relato do resultado de testagem para sífilis nos últimos 12 meses, em cinco capitais brasileiras, estudo TransOdara, 2019-2021 (n = 686)

Variáveis	Resultado positivo para sífilis nos últimos 12 meses n = 211 (%)	Resultado negativo para sífilis nos últimos 12 meses n = 475 (%)	p-valor
Características Sociodemográficas			
**Cidade**			0,391
São Paulo	96 (45,5)	203 (42,8)	
Porto Alegre	25 (11,9)	63 (13,3)	
Salvador	28 (13,3)	63 (13,3)	
Manaus	45 (21,3)	85 (17,9)	
Campo Grande	17 (8,1)	60 (12,7)	
Dados ausentes	0	1	
**Faixa etária (anos)**			0,051
18-24	34 (16,1)	123 (25,9)	
25-29	50 (23,7)	111 (23,4)	
30-39	76 (36,0)	135 (28,4)	
40-49	38 (18,0)	74 (15,6)	
50+	13 (6,2)	32 (6,7)	
**Identidade de g** **ênero**			0,808
Mulher	16 (7,6)	36 (7,6)	
Mulher trans	127 (60,2)	295 (62,1)	
Travesti	62 (29,4)	121 (25,5)	
Transexual	2 (1,0)	11 (2,3)	
Não binária	3 (1,4)	9 (1,9)	
Outras	1 (0,5)	2 (0,6)	
**Orientação sexual**			
Heterossexual	177 (84,3)	360 (76,3)	0,031
Homossexual	8 (3,8)	33 (7,0)	
Bissexual	17 (8,1)	30 (6,4)	
Pansexual	8 (3,8)	44 (9,3)	
Assexual	0	1 (0,2)	
Outras	0	4 (0,9)	
Dados ausentes	1	3	
**Raça/cor da pele**			
Branca	58 (27,8)	128 (27,1)	0,757
Preta	48 (23,0)	121 (25,6)	
Amarela	3 (1,4)	13 (2,8)	
Parda	96 (45,9)	201 (42,6)	
Indígena	4 (1,9)	9 (1,9)	
Dados ausentes	2	3	
**Escolaridade**			0,000
Ensino fundamental ou menos	70 (33,2)	89 (18,7)	
Ensino médio incompleto	37 (17,5)	78 (16,4)	
Ensino médio completo	73 (34,6)	182 (38,3)	
Ensino superior ou mais	31 (14,7)	126 (26,5)	
**Ocupação**			0,011
Emprego formal	57 (27,3)	136 (28,7)	
Emprego informal	43 (20,6)	125 (26,4)	
Desempregada	37 (17,7)	100 (21,1)	
Estudante	9 (4,3)	27 (5,7)	
Profissional do sexo	63 (30,1)	86 (18,1)	
Dados ausentes	2	1	
**Situação de moradia**			0,861
Próprio ou alugado	139 (65,9)	317 (66,7)	
Moradia temporária (família, amigos ou trabalho)	46 (21,8)	102 (21,5)	
Abrigo ou área livre	16 (7,6)	29 (6,1)	
Outro	10 (4,7)	27 (5,7)	
**Renda mensal**			0,328
Até 1 salário mínimo	106 (54,6)	228 (50,4)	
Acima do salário mínimo	88 (45,4)	224 (49,6)	
Dados ausentes	17	23	
**Encarceramento**			0,000
Não	133 (63,3)	373 (78,7)	
Sim	77 (36,7)	101 (21,3)	
Dados ausentes	1	1	
**Cuidado com a saúde**			
**Sorologia HIV**			0,000
Negativo ou desconhecido	138 (66,4)	376 (79,2)	
Positivo	70 (33,7)	99 (20,8)	
Dados ausentes	3	0	
**Consulta com profissional de saúde nos últimos 12 meses**			0,831
Não (sem necessidade)	32 (15,2)	64 (13,6)	
Não (tinha necessidade)	8 (3,8)	17 (3,6)	
Sim	170 (81,0)	391 (82,8)	
Dados ausentes	1	3	
**Testou para HIV nos últimos 12 meses**			0,695
Não	34 (16,1)	71 (14,9)	
Sim	177 (83,9)	404 (85,1)	
**Modificações corporais**			
**Uso de hormônios para transição de gênero**			0,24
Não	15 (7,1)	47 (9,9)	
Sim	196 (92,9)	428 (90,1)	
**Uso de silicone industrial**			0,001
Não	127 (60,2)	345 (73,2)	
Sim	84 (39,8)	126 (26,8)	
Dados ausentes	0	4	
**Cirurgia de afirmação de gênero**			0,663
Não	208 (98,6)	466 (98,1)	
Sim	3 (1,4)	9 (1,9)	
**Fatores sociais e interpessoais**			
**Discriminação nos últimos 12 meses**			0,837
Não	23 (11,5)	52 (10,9)	
Sim	185 (88,5)	423 (89,1)	
Dados ausentes	3	0	
**Maltratadas por profissionais da saúde**			0,791
Não	141 (67,3)	314 (66,1)	
Sim	69 (32,7)	161 (33,9)	
Dados ausentes	1	0	
**Agressão verbal, humilhação ou insulto nos últimos 12 meses**			0,968
Não	98 (47,1)	223 (46,9)	
Sim	110 (52,9)	252 (53,1)	
Dados ausentes	3	0	
**Agressão física nos últimos 12 meses**			0,064
Não	165 (78,6)	400 (84,4)	
Sim	45 (21,4)	74 (15,6)	
Dados ausentes	1	1	
**Comportamento sexual**			
**Trabalho sexual como fonte de renda no último mês**			0,004
Não	140 (66,3)	365 (76,8	
Sim	71 (33,7)	110 (23,2)	
**Fez sexo em troca de bens**			0,036
Não	114 (25,8)	128 (36,0)	
Sim	95 (74,2)	228 (64,0)	
Missing	2	119	
**Uso de aplicativos de relacionamento**			0,337
Não	143 (68,1)	299 (64,3)	
Sim	65 (31,9)	166 (35,7)	
Missing	3	10	
**Primeira relação sexual**			0,471
Não	33 (16,0)	85 (18,3)	
Sim	173 (84,0)	379 (81,7)	
Dados ausentes	5	11	

Em relação à moradia, 65,9% das pessoas relataram viver em casa ou apartamento próprio ou alugado e 54,6% tinham renda até 1 salário mínimo^13^ (em 2021, R$ 1.100,00). Quanto à raça/cor da pele, 45,9% se identificavam como pardas e 23,0% como pretas. A maior escolaridade foi ensino médio completo (para 34,6% da amostra), e o trabalho sexual foi indicado como ocupação principal para 30,1% da amostra. Cerca de um terço (36,7%) já havia sido encarcerada. No que diz respeito a cuidados de saúde, 81,0% consultaram um profissional de saúde nos últimos 12 meses, 83,9% fizeram o teste de HIV e 33,7% testaram positivo para o HIV. Quanto à modificação corporal, 92,6% usavam hormônios para a transição de gênero, 39,8% utilizavam silicone industrial e 1,4% passou por cirurgia genital para adequação ao gênero com o qual se identificam. Aproximadamente 90,0% relataram ter sofrido discriminação nos últimos 12 meses, 52,1% sofreram agressão verbal e 21,3% foram fisicamente agredidas por serem MTTr. Destaca-se que 32,7% relataram ter sido maltratadas por profissionais de saúde alguma vez. 

Houve diferença, com significância estatística, entre os grupos que relataram testagem positiva e negativa para sífilis nas seguintes variáveis: orientação sexual, escolaridade, ocupação, encarceramento, sorologia HIV, uso de silicone industrial, trabalho sexual como fonte de renda no último mês e fazer sexo em troca de bens. As demais características estão descritas na Tabela 1.

A Tabela 2 apresenta as prevalências das características sociodemográficas e comportamentais resultantes da análise bivariada (OR) e do modelo múltiplo ajustado (ORa) de cada um dos subgrupos com relato de testagem positiva (tratamento ausente/incompleto e tratamento completo).

**Tabela 2:** Análise bivariada e multivariada (OR) e (ORa), com intervalos de confiança de 95% (IC95%), associações independentes com completude autorrelatada do tratamento de sífilis entre mulheres trans e travestis em

Variáveis	Tratamento incompleto ou ausente	Tratamento completo	p-valor	OR (IC_95%_)	ORa (IC_95%_)
	n = 82 (38,9%)	n = 129 (61,1%)			
**Características sociodemográficas**					
**Cidade**							
São Paulo	55 (57,3)	41 (42,7)	< 0,001	ref.	ref.
Porto Alegre	5 (20,0)	20 (80,0)		5,37 (1,86;15,49)	5,89 (2,00;17,34)
Salvador	9 (32,1)	19 (67,9)		2,83 (1,16;6,90)	3,09 (1,24;7,66)
Manaus	8 (17,8)	37 (82,2)		6,20 (2,61;14,73)	6,25 (2,60;15,03)
Campo Grande	5 (29,4)	12 (70,6)		3,22 (1,05;9,86)	3,73 (1,19;11,70)
**Faixa etária (anos)**							
18-24	13 (38,2)	21 (61,8)	0,693		
25-29	20 (40,0)	30 (60,0)			
30-39	32 (42,1)	44 (57,9)			
40-49	11 (29,0)	27 (71,1)			
50+	6 (46,2)	7 (53,9)			
**Identidade de gênero**							
Mulher	9 (56,3)	7 (43,8)	0,276		
Mulher trans	52 (40,9)	75 (59,1)			
Travesti	21 (33,9)	41 (66,1)			
Transexual	0	2 (100,0)			
Não binária	0	3 (100,0)			
Outras	0	1 (100,0)			
**Orientação sexual**							
Heterossexual	73 (41,2)	104 (58,8)	0,589		
Homossexual	2 (25,0)	6 (75,0)			
Bissexual	5 (29,4)	12 (70,6)			
Pansexual	2 (25,0)	6 (75,0)			
Dados ausentes	0	1			
**Raça/cor da pele**							
Branca	22 (37,9)	36 (62,1)	0,986		
Preta	18 (37,5)	30 (62,5)			
Amarela	1 (33,3)	2 (66,7)			
Parda	38 (39,6)	58 (60,4)			
Indígena	2 (50,0)	2 (50,0)			
Dados ausentes	1	1			
**Escolaridade**					
Ensino fundamental ou menos	25 (35,7)	45 (64,3)	0,498		
Ensino médio incompleto	18 (48,7)	19 (51,4)			
Ensino médio completo	29 (39,7)	44 (60,3)			
Ensino superior ou mais	10 (32,3)	21 (67,8)			
**Ocupação**					
Emprego formal	19 (33,3)	38 (66,7)	0,105	ref.	
Emprego informal	13 (33,3)	26 (66,7)		1,00 (0,42;2,37)	
Desempregada	13 (31,7)	28 (68,3)		1,08 (0,46;2,54)	
Estudante	6 (66,7)	3 (33,3)		0,25 (0,06;1,11)	
Profissional do sexo	31 (49,2)	32 (50,8)		0,52 (0,25;1,08)	
Dados ausentes	0	2			
**Situação de moradia**						
Própria ou alugada	60 (43,2)	79 (56,8)			
Moradia temporária (família, amigos ou trabalho)	14 (29,8)	33 (70,2)	0,307		
Abrigo ou situação de área livre	6 (37,5)	10 (62,5)			
Outro	2 (22,2)	7 (77,8)			
**Renda mensal** ^a^						
Até 1 salário mínimo	39 (36,8)	67 (63,2)	0,456		
Acima do salário mínimo	37 (42,1)	51 (58,0)			
Dados ausentes	6	11			
**Encarceramento** ^b^						
Não	52 (39,1)	81 (60,9)	0,837		
Sim	29 (37,7)	48 (62,3)			
Dados ausentes	1	0			
**Cuidados de saúde**						
**Sorologia HIV**						
Negativo ou desconhecido	61 (44,2)	77 (55,8)	0,029	ref.
Positivo	20 (28,6)	50 (71,4)		1,98 (1,07;3,67)
Dados ausentes	1	2			
**Consulta com profissional de saúde** **nos últimos 12 meses**						
Não (sem necessidade)	15 (46,9)	17 (53,1)	0,479		
Não (tinha necessidade)	2 (25,0)	6 (75,0)			
Sim	64 (37,7)	106 (62,4)			
Dados ausentes	1	0			
**Testou para HIV nos últimos 12 meses**						
Não	7 (20,6)	27 (79,4)	0,017	ref.
Sim	75 (42,4)	102 (57,6)		0,35 (0,15;0,85)
**Modificações corporais**						
**Uso de hormônios para transição de gênero**						
Não	6 (40,0)	9 (60,0)	0,925		
Sim	76 (38,8)	120 (61,2)			
**Uso de silicone industrial**						
Não	43 (33,9)	84 (66,1)	0,067	ref.
Sim	39 (46,4)	45 (53,6)		0,59 (0,34;1,04)
**Cirurgia de afirmação de gênero**						
Não	82 (39,4)	126 (60,6)	0,164		
Sim	0	3 (100,0)			
**Fatores sociais e interpessoais**						
**Foi discriminada últimos 12 meses**						
Não	12 (46,2)	14 (53,9)	0,415		
Sim	70 (37,8)	115 (62,2)			
**Já foi maltratada por profissionais da saúde**						
Não	55 (38,7)	87 (61,3)	0,956		
Sim	27 (39,1)	42 (60,9)			
**Foi verbalmente agredida, humilhada** **ou insultada nos últimos 12 meses**							
Não	31 (30,7)	70 (69,3)	0,020	ref.	ref.
Sim	51 (46,4)	59 (53,6)		0,51 (0,29;0,90)	0,46 (0,25;0,85)
**Foi fisicamente agredida nos últimos 12 meses**							
Não	61 (36,8)	105 (63,3)	0,226		
Sim	21 (46,7)	24 (53,3)			
**Comportamento sexual**							
**Trabalho sexual como fonte de renda no** **último mês**							
Não	50 (61,0)	90 (69,8)	0,188		
Sim	32 (45,1)	39 (54,9)			
**Fez sexo em troca de dinheiro, bens,** **drogas, ou de um local para morar**							
Não	51 (44,7)	63 (55,3)	0,074	ref.
Sim	31 (32,6)	64 (67,4)		1,67 (0,95;2,94)
Dados ausentes	0	2			
**Uso de aplicativos de relacionamento**							
Não	57 (39,9)	86 (60,1)	0,539		
Sim	23 (35,4)	42 (64,6)			
Dados ausentes	2	1			
**Primeira relação sexual consentida**							
Não	13 (39,4)	20 (60,6)	0,992		
Sim	68 (39,3)	105 (60,7)			
Dados ausentes	1	2			

Os seguintes fatores foram associados ao desfecho na análise bivariada: ter testado para HIV e ter testado positivo para HIV nos últimos 12 meses, cidade de recrutamento e experiência de agressão verbal por ser uma mulher trans. Participantes em São Paulo relataram níveis substancialmente mais baixos de completude em comparação com outras cidades (São Paulo, 42,7%; Salvador, 67,9%; Campo Grande, 70,6%; Porto Alegre, 80,0%; e Manaus, 82,7%, p < 0,001). 

MTTr que reportavam teste de HIV positivo tinham maior probabilidade de completar o tratamento em comparação com aquelas que relataram um teste HIV negativo ou *status* desconhecido (71,4% vs. 55,8%, p = 0,029). MTTr testadas para HIV nos últimos 12 meses tiveram menor probabilidade de completar tratamento para sífilis em comparação com aquelas que não fizeram o teste (57,6% *vs*. 79,4%, p = 0,017).

Com base no modelo múltiplo ajustado, apenas a cidade do estudo e a experiência anterior de agressão verbal permaneceram associadas à incompletude/ausência do tratamento de sífilis. Comparadas às MTTr em São Paulo, as participantes das demais cidades apresentaram taxas de completude maiores, variando de ORa 3,09 (IC_95_% 1,24;7,66), em Salvador, a ORa 6,25 (IC_95_% 2,60;15,03) em Manaus. MTTr que foram verbalmente agredidas, humilhadas ou insultadas por serem MTTr, nos últimos 12 meses, tiveram menor probabilidade de completar o tratamento (ORa 0,46; IC_95_% 0,25;0,85).

## DISCUSSÃO

O estudo revelou um preocupante histórico de sífilis entre MTTr, agravado pela baixa adesão ao tratamento. Mais de um terço das participantes com diagnóstico prévio de sífilis relataram não ter iniciado ou não ter concluído o tratamento. Esse elevado resultado de ausência/incompletude do tratamento é semelhante ao encontrado em Lima, no Peru, onde 31,3% das MTTr e homens que fazem sexo com homens (HSH), após testes positivos para sífilis, enfrentaram desafios semelhantes.^14^

Em relação aos fatores associados, surpreendentemente, as participantes de São Paulo apresentaram a menor taxa de completude do tratamento, apesar de essa ser a maior cidade do país e estar localizada na região Sudeste, que é relativamente mais rica. Por outro lado, em Manaus, mesmo com recursos financeiros mais limitados, as participantes apresentaram o maior nível de adesão ao tratamento. Acreditamos que a existência de um ambulatório especializado em diversidade sexual e de gênero em Manaus, que facilita o acesso à saúde para essa população, contribua para explicar a taxa mais alta na cidade em comparação com São Paulo. Embora São Paulo possua uma rede de saúde mais ampla, apresenta experiências heterogêneas no tratamento às pessoas trans, o que pode influenciar nas diferentes taxas de conclusão do tratamento de Sífilis.

É importante destacar que a experiência de agressão verbal, especialmente para MTTr, está associada a uma menor probabilidade de conclusão do tratamento para sífilis. Isso ocorre porque as MTTr frequentemente enfrentam altos níveis de violência interpessoal de diversos tipos.^15^ A agressão verbal pode ser um indicador da extensa discriminação enfrentada pelas MTTr no Brasil;^16-18^ além disso, é amplamente documentado que MTTr podem evitar assistência em saúde por medo de discriminação no SUS.^8,9^ A hesitação em utilizar serviços de saúde para obter diagnóstico e tratamento para sífilis e outras IST entre MTTr é uma estratégia adotada por elas para evitar o estigma que frequentemente ocorre nesses ambientes.^9^

Outra hipótese plausível para a baixa adesão ao tratamento, durante o período analisado, pode estar relacionada à pandemia de covid-19, que impactou o acesso aos serviços de saúde para grupos vulneráveis, incluindo MTTr.^19^

A pandemia de covid-19, apesar de gerar atraso na realização das entrevistas, devido à suspensão dos campos de atuação, em razão de toques de recolher e de medidas para diminuir a circulação de pessoas durante o período de restrições, em todas as cidades da pesquisa, não reduziu a capacidade de atingirmos e até ultrapassarmos a amostra inicialmente calculada.

Barreiras ao tratamento de sífilis entre MTTr estão presentes em todas as etapas da cascata de cuidados. Para confirmar o diagnóstico, são necessários dois testes, um treponêmico e outro não treponêmico, que em geral não são realizados concomitantemente.^6^

Embora atualmente se recomende que o tratamento de pessoas pertencentes a grupos em situação de maior vulnerabilidade seja iniciado imediatamente após o resultado positivo do teste rápido, é possível que essa orientação não seja de conhecimento ou seguida por todos(as) os(as) profissionais de saúde.^6^ Além disso, a complexidade do tratamento também pode ser uma barreira, haja vista que, no diagnóstico de sífilis tardia, são necessárias injeções de penicilina em intervalos de sete dias, ou ainda o tratamento oral com doxiciclina, que precisa ser mantido por um mês.^6^

Outro fator que influencia na cascata de cuidados é a dificuldade de rastreamento de parcerias sexuais para tratamento, e dificuldade na negociação de uso de preservativos como método de prevenção com parcerias comerciais, como documentado em grupos de HSH.^20^

Adicionalmente, o uso de silicone industrial líquido, prática ainda frequente nesse grupo,^21^ dificulta a aplicação intramuscular da penicilina. Isso ocorre porque é necessário injetar em locais alternativos, como o ventroglúteo, com os quais os profissionais de saúde estão menos habituados.^7^ O tratamento alternativo com doxiciclina, por outro lado, utilizado como alternativa ao injetável, e frequentemente indicado para MTTr com silicone industrial, pode resultar em abandono de tratamento, devido à baixa tolerabilidade por causa dos efeitos colaterais (gastrointestinais), tempo de tratamento prolongado, posologia (duas vezes ao dia) e possíveis interações medicamentosas.^6^

Outros fatores estruturais podem contribuir para a dificuldade de MTTr serem tratadas de forma apropriada, e incluem a falta de acessibilidade, serviços não acolhedores,^8-10^ por serem vítimas de assédio no transporte,^22^ por falta de recursos para o transporte^23^ ou mesmo o horário de funcionamento dos serviços,^23^ incompatível com o horário de trabalho de uma parte delas. 

As diferentes proporções de incompletude/ausência do tratamento entre as cidades instigam a necessidade de estudos mais detalhados, que investiguem as razões para esses achados, de modo a serem orientadas ações para mitigar a não completude do tratamento e reduzir as altas prevalências de sífilis nessa população.

Profissionais de saúde, gestores, equipes multiprofissionais e sociedade civil no Brasil precisam estar mais sensibilizados para o cuidado das pessoas trans e travestis, e envolvidos na redução das barreiras que elas encontram no sistema de saúde. Isso é especialmente válido para a saúde sexual, comportamentos de risco e triagem para sífilis e outras ISTs. Embora na amostra a incompletude/ausência do tratamento para sífilis não tenha sido associada à agressão pelos profissionais de saúde, isso foi relatado por quase um terço das participantes no último ano, o que corrobora a literatura.^24^

O presente estudo apresenta várias limitações. Primeiramente, os dados foram obtidos por meio de autorrelato, sujeito a viés de memória. Em segundo lugar, os dados não são representativos do total de MTTr no Brasil. Apesar de o RDS ser amplamente utilizado para estudar populações consideradas de difícil acesso, é importante interpretar esses resultados com cautela, pois podem ser representativos apenas das redes sociais capturadas pelo estudo em cada uma das cidades estudadas, não incluindo redes de outros estados, ou cidades menores do mesmo estado. Apesar das limitações citadas, o estudo recrutou um número expressivo de MTTr nas diversas regiões do Brasil, e seus achados colaboram com a crescente literatura sobre sífilis em mulheres trans e travestis, sendo um dos poucos que estudou o tratamento de sífilis neste grupo populacional.

A triagem e o tratamento de rotina para sífilis precisam ser integrados aos programas de saúde em todo o país. Novas tecnologias, como métodos diagnósticos moleculares, autoteste,^25^ estratégias baseadas em pares^26^ e serviços de saúde com horários flexíveis,^23^ podem ajudar a mitigar essas barreiras, sendo necessário incluir a avaliação da efetividade dessas diferentes opções. Além disso, os profissionais de saúde devem estar cientes da legislação e dos protocolos que já foram estabelecidos para atender às necessidades dessa população, incluindo o uso do nome social de MTTr em ambientes de saúde, a aplicação de medicação injetável em sítios alternativos e o estabelecimento de mecanismos para prevenir e reparar danos causados pela discriminação que as MTTr enfrentam na assistência à saúde.^9^
